# PD-L1 Expression and Tumour Microenvironment Patterns in Resected Non-Small-Cell Lung Cancer

**DOI:** 10.3390/medicina60030482

**Published:** 2024-03-14

**Authors:** Giedrė Gurevičienė, Jurgita Matulionė, Lina Poškienė, Skaidrius Miliauskas, Marius Žemaitis

**Affiliations:** 1 Department of Pulmonology, Medical Academy, Lithuanian University of Health Sciences, 44307 Kaunas, Lithuania; 2 Department of Pathology, Medical Academy, Lithuanian University of Health Sciences, 44307 Kaunas, Lithuania

**Keywords:** programmed death-ligand 1, tumour microenvironment, non-small cell lung cancer

## Abstract

*Background and Objectives*: Although perioperative immunotherapy is implemented as a standard of care for resected non-small cell lung cancer (NSCLC), there is unmet need for predictive biomarkers as programmed death-ligand 1 (PD-L1) is not the perfect one. The functionality of tumour-infiltrating immune cells in the tumour microenvironment (TME) and the involvement in immune system response is one of the crucial factors that lead to pro- or anti-tumourigenic role and could predict response to PD-1 and PD-L1 inhibitors. So, the investigation of PD-L1 expression in the context of TME in early stages of resected NSCLC is urgent required. *Materials and Methods*: PD-L1 expression by three scoring methods: tumour proportion score (TPS), immune cell score (IC), and combined proportion score (CPS) was assessed in 72 archival tumour tissue specimens from stage I–III surgically resected NSCLC patients and associations with immune cells in TME were explored. *Results*: PD-L1 expression ≥1% evaluated by TPS, IC, and CPS was detected in 28%, 36%, and 39% of cases and moderate, substantial, and strong agreement between TPS and IC, TPS and CPS, CPS and IC was detected (Cohen’s κ coefficient 0.556, 0.63, and 0.941, respectively). PD-L1 TPS, IC, and CPS correlated with smoking intensity defined as pack-years (r = 0.0305, *p* = 0.012; r = 0.305, *p* = 0.013, and r = 0.378, *p* = 0.002, respectively). Only PD-L1 TPS was associated with squamous cell carcinoma (*p* = 0.028). PD-L1 IC ≥1% was more often seen in tumours with high CD4^+^ T cells infiltration (*p* = 0.02), while PD-L1 CPS ≥1%—in tumours with high CD4^+^ and CD8^+^ T cells infiltration (*p* = 0.021 and *p* = 0.048, respectively). PD-L1 IC and CPS ≥10% was more often detected in tumours with greater number of tumour-infiltrating CD4^+^Foxp3^+^ T cells (*p* = 0.01 and *p* = 0.025, respectively). PD-L1 TPS ≥50% was associated with higher probability to detect greater number of tumour-infiltrating M2 macrophages (*p* = 0.021). No association was found between PD-L1 alone or in combination with tumour-infiltrating lymphocytes, macrophages, and disease-free or overall survival. *Conclusions*: This study results revealed that rates of PD-L1 expression correlated among three scoring methods (TPS, IC, and CPS). Moreover, PD-L1 expression was significantly associated with smoking intensity, squamous histology, and tumour-infiltrating immune cells.

## 1. Introduction

Lung cancer remains one of the most common oncological diseases and leading cause of cancer-related death worldwide (2.21 m. death annually) [1]. Stage is one of the most important prognostic factors in non-small cell lung cancer (NSCLC) with better prognosis in early lung cancer [2]. For metastatic or locally advanced NSCLC, immunotherapy alone or in combination with chemotherapy, or chemoradiotherapy, became a standard of treatment [3,4]. More recently immunotherapy was tested and/or approved as adjuvant, neoadjuvant, or perioperative approach in resected early NSCLC [2,5,6,7,8]. Although a breakthrough in NSCLC immunotherapy was made, the benefit is still modest with 15%–30% of response rates [9,10], and 13–32% of 5 year survival for advanced NSCLC [11]. The pathological complete response and major pathological response range from 15% to 83%, respectively, in resected NSCLC with neoadjuvant immunotherapy [12,13]. Although correlation between PD-L1 expression and responses to immunotherapy was established, PD-L1 is not a perfect predictive marker. In clinical studies of advanced NSCLC, from 55% to 75% of patients with high PD-L1 expression did not respond to immunotherapy, whereas 10% of patients with negative PD-L1 expression responded [4,11,14]. The clinical trial Impower010 showed that adjuvant atezolizumab improved overall survival (OS) compared with best supportive care in stage II–IIIA resected NSCLC with high PD-L1 expression, but the PEARLS/KEYNOTE-091 trial did not find a statistically significant benefit of adjuvant pembrolizumab in this setting [6,7].

Recent research suggests that the response to immunotherapy is related not only with PD-L1 expression, but also with other factors, such as molecular and genomic profiling immune cells response in tumour microenvironment (TME) and others [15,16,17,18,19]. The functionality of tumour-infiltrating immune cells in the TME and the involvement in immune system response is one of the crucial factors that lead to pro- or anti-tumourigenic role and could predict response to PD-1 and PD-L1 inhibitors [18,19]. While most of PD-1/PD-L1 inhibitors were first approved in the treatment of advanced NSCLC, the greater part of studies paid attention to more detailed investigation of PD-L1 expression and TME association in these patients [18,20,21,22]. Currently, limited research exists on early resected NSCLC, and the results are controversial [23,24]. For example, in early-stage NSCLC, compared to advanced disease, an association between PD-L1 expression and CD4^+^Foxp3^+^ T cells, CD8^+^ T cells and M2 macrophages, but not CD4^+^ T cells were detected [23,25,26]. Moreover, clinical data revealed discordance in the correlation between PD-L1 expression and prognosis within TME [18,22,23].

Furthermore, several diagnostic immunohistochemistry (IHC) assays with different scoring systems were approved for PD-L1 testing in NSCLC. For example, most of PD-L1 IHC assays, such as Dako 22C3 or Dako 28-8, are based on scoring PD-L1 expression only on tumour cells as tumour proportion score (TPS) [27]. Exclusively, the Ventana PD-L1 SP142 assay is approved by US Food and Drug Administration (FDA) as well as European Medicines Agency (EMA) for testing PD-L1 expression on tumour cells (TC) and immune cells (IC) [28]. What is more, in most of other malignancies, such as cervical cancer, urothelial carcinoma, head and neck squamous cell carcinoma, oesophageal carcinoma, gastric or gastro-oesophageal junction adenocarcinoma, approval for monotherapy or combined therapy is based on scoring PD-L1 expression on tumour and immune cells together by combined proportion score (CPS) [29]. In contrast, PD-L1 expression on immune cells can be the main and only area of interest for the determination of immunotherapy. For example, in triple-negative breast cancer and urothelial carcinoma, approval for atezolizumab monotherapy or combined therapy is given in concern of PD-L1 expression evaluated on immune cells only as percent of tumour area occupied by immune cells [29,30]. These observations suggest that the PD-L1 expression not only on tumour cells, but also on immune cells and both on tumour and immune cells together with TME attempts to play an important role in regulating the anti-tumour response. Extended studies of PD-L1 expression in context of TME in early stages of NSCLC are required.

The aim of this study was to evaluate PD-L1 expression on tumour cells and immune cells using three scoring methods (TPS, IC, and CPS) and to analyse the relationship of these results with TME and the possible prognosis of early-stage NSCLC patients who underwent surgical resection.

## 2. Materials and Methods

### 2.1. Methods

Ethical approval for this research protocol was obtained by Kaunas Regional Ethics Committee for Biomedical Research (No. BE-2-44).

### 2.2. Study Population

Lung tissue specimens of 72 cases were selected from the archive of patients, who were diagnosed with stage I–III NSCLC and underwent surgical resection at the Hospital of Lithuanian University of Health Sciences, Kaunas Clinics between September 2012 and February 2015. These tumour specimens were already assessed for tumour-infiltrating lymphocytes (TILs) and tumour-infiltrating macrophages (TAMs) [31,32,33].

Tumours were staged according to the 7th edition of the TNM Classification of Malignant Tumours [34]. The histology of lung tumours was classified according to the World Health Organization (WHO) classification of lung tumours [35]. None of the patients underwent prior anti-PD-1/PD-L1 therapy, EGFR/ALK-targeted therapy, neoadjuvant chemotherapy, or radiotherapy. However, patients with stage II/III disease may have been offered adjuvant chemotherapy or radiotherapy. All patients’ clinical and pathological information was collected from the medical documentation: clinical records and pathology reports. The diagnosis of chronic obstructive pulmonary disease (COPD) was based on the Global Initiative for Chronic Obstructive Lung Disease (GOLD) criteria [36].

Exclusion criteria encompassed a history of additional malignancies, connective tissue diseases, any unstable systemic conditions (including active infections and significant cardiovascular disease), along with instances of COPD exacerbation and systemic glucocorticoid therapy within the month preceding the surgery.

Smoking status was based on cigarettes smoked in their lifetime—patients who have smoked more than 100 cigarettes in their lifetime were defined as smokers (current or former), otherwise they were considered to be non-smokers. Pack-years (the number of cigarette packs patient smoked per day multiplied by the number of years) were used to quantify smoking history.

### 2.3. Immunohistochemistry Analysis

Murine monoclonal anti-PD-L1 antibody, clone 22C3 pharmDx (Agilent Technologies/Dako, Carpinteria, CA, USA), was used for PD-L1 staining. The IHC staining procedure was performed on a Roche Ventana Benchmark XT automated slide stainer (Ventana Medical Systems, Roche, France), following manufacturer’s instructions. Tissue sections from 3 to 5 µm thick from the formalin-fixed, parafin-embedded (FFPE) tissue of NSCLC were cut, subsequently de-waxed and rehydrated through graded alcohols. Positive control of human tonsil and placenta was used for each slide. PD-L1 IHC was evaluated and scored by two investigators (G.G. and L.P), under light microscope (Olympus BX50 microscope (Olympus Co., Tokyo, Japan)). Any disagreement and difficult cases were determined by discussing and reviewing the specimen, and consensus decision was made.

First hematoxylin and eosin stained slides were assessed to evaluate staining quality and preservation. Further, the control tissue was examined. Human tonsil (PD-L1 membrane staining in the crypt epithelium and macrophages within the lymphoid follicles) and placenta (PD-L1 staining observed in syncitiotrophoblastic cells) were used as positive control tissue [37,38]. Assessing slides for PD-L1 expression, necrotic cells or degenerated tumour cells were excluded. Each slide included at least 100 viable tumour cells suitable for evaluation. PD-L1 expression was assessed by three scoring methods.

PD-L1 expression on tumour cells was evaluated by TPS—the percentage of viable tumour cells with complete circumferential or partial linear expression at any intensity of PD-L1 relative to all viable tumour cells in the examined section [38]. Based on data from previous clinical trials, cases were considered positive for PD-L1 when ≥1% of the tumour cells expressed PD-L1; otherwise, they were considered as negative. Furthermore, the level of PD-L1 defined by TPS was categorised into three subgroups: no expression (<1%), low expression (1–49%), and high expression (≥50%) [4].

PD-L1 expression on immune cells was evaluated by tumour-infiltrating IC—the proportion of tumour area occupied by PD-L1 positive immune cells (lymphocytes and macrophages) with membrane and/or cytoplasmic PD-L1 staining at any intensity. Cases were considered positive when immune cells expressing PD-L1 occupied ≥1% of tumour area, otherwise they were considered as negative. Furthermore, the level of PD-L1 defined by IC was categorised into three subgroups: no expression (<1%), low expression (1–10%), and high expression (≥10%) [3].

Finally, PD-L1 expression on tumour cells and immune cells was evaluated by CPS—the sum of PD-L1 positive tumour cells and immune cells (lymphocytes and macrophages) divided by the total number of viable tumour cells and multiplied by 100. Cases were considered positive for PD-L1 when ≥1% of the tumour and/or immune cells expressed PD-L1, otherwise they were considered as negative. Furthermore, the level of PD-L1 defined by CPS was categorised into three subgroups: no expression (<1%), low expression (1–10%), and high expression (≥10%) [3].

Scoring of CD4^+^ T cells, CD8^+^ T cells, Foxp3^+^CD4^+^ T cells, interleukin (IL)-17A^+^CD4^+^ T cells, M1 and M2 macrophages has been previously reported [31,32,33].

### 2.4. Statistical Analysis

Statistical analysis was performed using the Statistical Package for the Social Sciences (IBM SPSS Statistics Chicago, IL, USA), version 25. Sample normality was assessed by the Kolmogorov–Smirnov test. Median with minimal and maximal values was used for data that was not normally distributed. To estimate the differences between two independent variables Mann–Whitney U test was used. Additionally, the differences between more than two independent variables were assessed using Kruskal–Wallis test. Chi-square (χ^2^) test or Fisher’s exact test was used to analyse categorical data. Moreover, correlation was assessed by the Spearman rank test for continuous variables. For the agreement the analysis Cohen’s kappa coefficient was used. Overall survival was calculated as the duration between initial NSCLC diagnosis and the time of death from any cause, or last follow up, if the patient was still alive. Disease free survival was defined as the time from the initial NSCLC diagnosis until disease recurrence. Kaplan–Meier method was used for survival estimate and Cox proportional hazard model was used for multivariate survival analyses. Value of *p* < 0.05 was considered statistically significant.

## 3. Results

PD-L1 expression was assessed in 72 cases of stage I–III NSCLC patients who underwent surgical resection. Demographic and clinical characteristics of study patients are presented in Table 1. Briefly, the mean age of the patients was 64.6 years, a significant portion of the population consisted of males who were current or former smokers, and one–third were diagnosed with COPD. The most common histological type was adenocarcinoma, and stage I–II were the most common postoperative pathological stages. Approximately half of the patients (43%) had received adjuvant chemotherapy and/or radiotherapy after surgery.

Considering PD-L1 expression evaluated by TPS, 5 (6.9%) cases demonstrated high PD-L1 expression, 14 (19.4%) cases demonstrated low PD-L1 expression, and 52 (72.2%) cases had no PD-L1 expression. Examining PD-L1 expression evaluated by IC 12 (16.7%) cases had high PD-L1 expression, 15 (20.5%) cases had low PD-L1 expression, and 46 (63.9%) cases had no PD-L1 expression on immune cells. Evaluating PD-L1 expression by CPS in 18 (25%) cases there was high PD-L1 expression, in 10 (13.9%) cases there was low PD-L1 expression, and in 44 (61.1%) cases there was no PD-L1 expression.

In the entire cohort, positive PD-L1 expression was seen in 20 (27.8%) cases evaluated by TPS, 26 (36.1%) cases evaluated by IC, and 28 (38.9%) cases evaluated by CPS.

PD-L1 TPS correlated with PD-L1 IC as well as PD-L1 CPS (r = 0.585, *p* = 0.001, and r = 0.759, *p* = 0.001, accordingly). There was moderate agreement between TPS and IC (Cohen’s kappa coefficient 0.556), substantial agreement between TPS and CPS (Cohen’s kappa coefficient 0.63), and strong agreement between CPS and IC (Cohen’s kappa coefficient 0.941), when PD-L1 expression was evaluated as positive or negative.

Association between PD-L1 expression and clinicopathological data are presented in Table 2. PD-L1 TPS was significantly associated with lung cancer histology subtype. More patients with squamous cell carcinoma had positive PD-L1 expression defined as TPS, compared to patients with adenocarcinoma. However, there was no association between histology and PD-L1 IC or CPS (Table 2).

**Figure 1:**
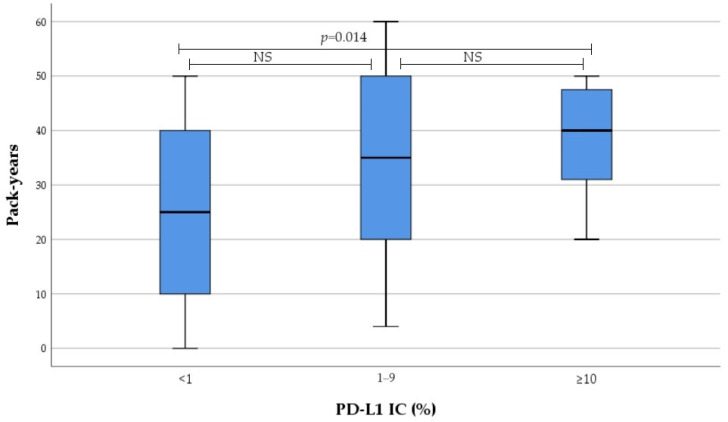
Association between PD-L1 IC and smoking intensity. *NS* not significant.

**Figure 2:**
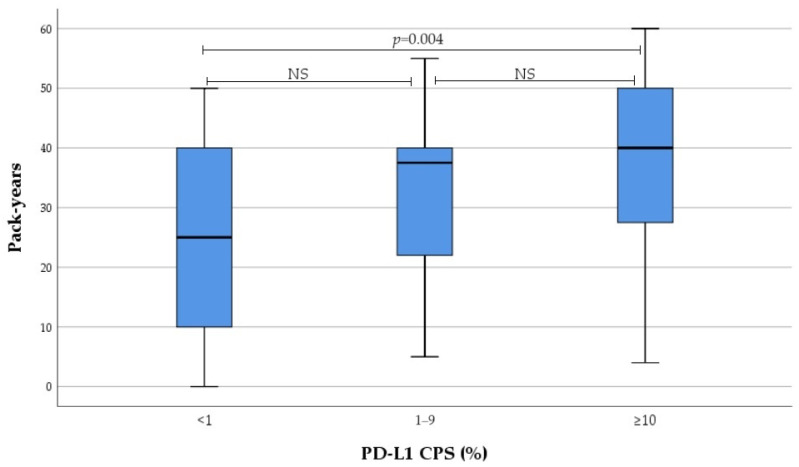
Association between PD-L1 CPS and smoking intensity. *NS* not significant.

Smoking intensity in current and former smokers gradually increased from no to high PD-L1 expression subgroup, as evaluated by IC and CPS. These differences were reached due to PD-L1 high expressors, with no differences observed between low and non-expressors (Figure 1 and Figure 2). Subsequent analysis revealed significant correlation between smoking intensity defined as pack-years and PD-L1 TPS (r = 0.0305, *p* = 0.012), IC (r = 0.305, *p* = 0.013), and CPS (r = 0.378, *p* = 0.002).

No significant association was found between PD-L1 TPS, IC, or CPS and patients’ age, tumour differentiation, tumour size, lymph node status, stage of the disease, or diagnosis of COPD (Table 2).

Association between PD-L1 expression subgroup and immune cells infiltration level in tumour tissue is presented in Table 3. Positive PD-L1 IC was more often seen in patients with high CD4^+^ T cells infiltration level in tumour tissue (*p* = 0.02). Moreover, positive PD-L1 CPS was more often detected in patients with high CD4^+^ T cells and high CD8^+^T cells infiltration level in tumour tissue (*p* = 0.021 and *p* = 0.048).

Furthermore, we analysed the subgroup with high PD-L1 expression in comparison to those with non-high PD-L1 expression, examining their associations with infiltration level of immune cells in tumour tissue. PD-L1 IC ≥ 10% and CPS ≥ 10% was detected significantly more often in patients with high Foxp3^+^CD4^+^ T cells infiltration level in tumour tissue (100% 10/10 and 77.8% 14/18, *p* = 0.002, respectively) compared to patients with low Foxp3^+^CD4^+^ T cells infiltration level (48.4% 30/62, and 48.1% 26/64, *p* = 0.032, respectively). No other associations were found.

We subsequently evaluated association between the number of tumour-infiltrating immune cells and PD-L1 expression subgroup. Patients were divided into subgroups taking as a threshold PD-L1 expression: ≥1% evaluated by TPS, IC and CPS (Table 4); ≥50% evaluated by TPS and ≥10%—by IC and CPS (Table 5). A significant association between PD-L1 expression subgroup and the number of tumour-infiltrating CD4^+^ T cells, Foxp3^+^CD4^+^ T cells, and M2 macrophages was found. Meanwhile, there was no association between PD-L1 expression subgroup and the number of tumour-infiltrating CD8^+^ T cells, IL-17A^+^CD4^+^ T cells, and M1 macrophages (Table 4 and Table 5).

**Figure 3:**
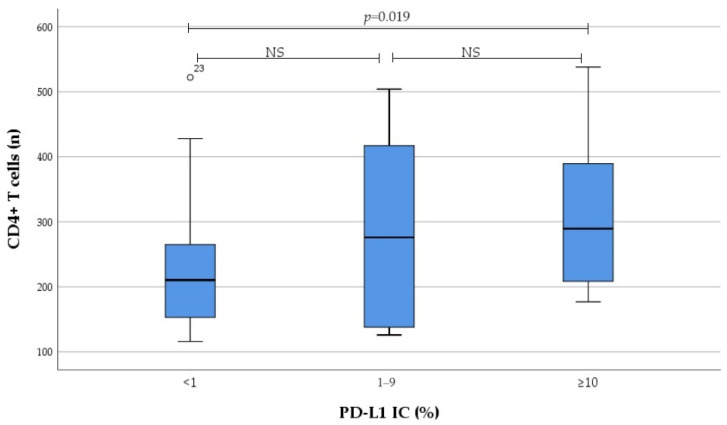
Association between PD-L1 IC subgroup and the number of tumour-infiltrating CD4^+^ T cells. *NS* not significant.

**Figure 4:**
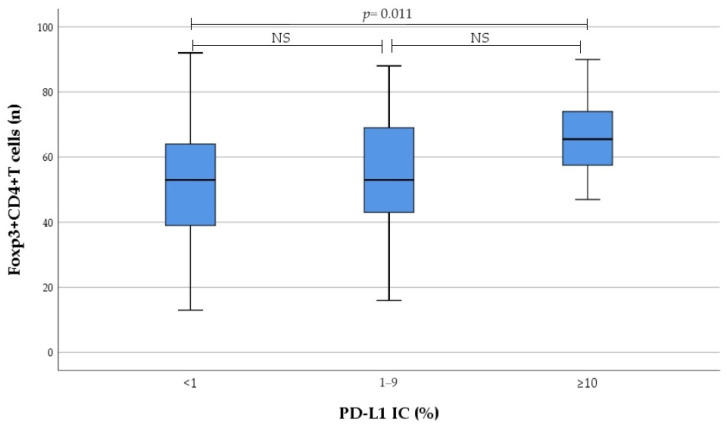
Association between PD-L1 IC subgroup and the number of tumour-infiltrating Foxp3^+^CD4^+^ T cells. *NS* not significant.

Results on the number of tumour-infiltrating immune cells regarding PD-L1 expression categorised to no, low, and high expression are presented in Figure 3 and Figure 4. We found that infiltration of CD4^+^ T cells, as well as Foxp3^+^CD4^+^ T cells in tumour tissue, gradually increased from no to high PD-L1 IC; this increase was primarily driven by the high PD-L1 expression group.

Eventually there was significant correlation between PD-L1 IC and the number of tumour-infiltrating CD4^+^ T cells (r = 0.279, *p* = 0.018), as well as Foxp3^+^CD4^+^ T cells (r = 0.251, *p* = 0.034). Moreover, significant correlation between PD-L1 CPS and the number of tumour-infiltrating CD4^+^ T cells (r = 0.280, *p* = 0.017) was detected. In contrast, no correlation was found between PD-L1 expression and the number of tumour-infiltrating IL17A^+^CD4^+^ T cells, as well as M1 and M2 macrophages.

Evaluating PD-L1 in relation with TME and disease-free survival, as well as overall survival, no significant association was found.

## 4. Discussion

The goal of this study was to evaluate PD-L1 expression in tumour tissue by three scoring methods (TPS, IC, and CPS) in context of TME and to evaluate PD-L1 expression as the prognostic value in early-stage resected NSCLC.

In this study, PD-L1 expression scores of TPS < 1%, 1% to 49% and ≥50% were observed in 72.2%, 20.8%, and 6.9% of cases. On the other hand, PD-L1 scores of IC < 1%, 1% to 10% and ≥10% were observed in 63.9%, 20.5%, and 16.7% of cases. Whereas PD-L1 expression scores of CPS < 1%, 1% to 10%, and ≥10% were observed in 61.1%, 13.9%, and 25% of cases among stage I–III resected NSCLC patients.

Distribution of PD-L1 expression in advanced NSCLC evaluated by TPS or CPS is broadly investigated, but information about real world distribution of PD-L1 expression in early stage NSLCL is limited. Previous clinical studies containing advanced NSCLC patients demonstrated that PD-L1 expression evaluated by TPS or CPS usually distributes as one third in the TPS < 1% group, one third in the 1% to 49% group, and one third in the ≥50% group [39,40]. Similar result were seen in randomised multicentre phase III clinical trials, where early stage resected NSCLC patients were included [6,7,41]. However, real-world experience demonstrates different results, with lower percentages of positive and high PD-L1 expression evaluated by TPS. In real-world retrospective study involving 211 cases of stage I–IV NSCLC, the prevalence of PD-L1 TPS ≥ 1% varied from 27% to 47.4% depending on the assay, while TPS ≥ 50% reached only 12.8% [42]. In another real-world unselected consecutive patient study, involving 800 cases of stage I–IV NSCLC, histological specimens were evaluated including cytology, biopsy, and resected samples, 63% of cases had positive TPS (≥1%) and 30% of cases had high TPS (≥50%). PD-L1 expression varied by the stage: TPS < 1%, 1% to 49% and ≥50% was 46%, 39% and 16% in stage I; 41%, 34%, and 25% in stage II; 35%, 37%, and 28% in stage III; 33%, 27%, and 40% in stage IV NSCLC. The lower stage was significantly associated with lower prevalence of PD-L1 positivity (odds ratio of 0.31 for stage I vs. stage IV) [43].

The results of this study regarding PD-L1 expression assessed by TPS align with findings of a study by Lin et al. where PD-L1 expression by TPS ≥ 50%, 1% to 49%, and <1% was seen in 10.6%, 24.7%, and 64.7% cases, respectively, when evaluating 170 archival surgically resected NSCLC samples [24]. Other larger retrospective study where majority of resected NSCLC specimens were stage I–IIIA (785/800), reported PD-L1 TPS <1%, 1% to 49% and ≥50% was in 75.3%, 17.5%, and 7.2% cases [44].

To our knowledge, this is the first study where PD-L1 expression was assessed by CPS and IC in early-stage NSCLC. Interestingly, when PD-L1 was evaluated by CPS in advanced NSCLC, higher positivity rates were seen compared to TPS. What is more, the greater part of positivity was reached due to IC positivity. The study of advanced NSCLC that evaluated PD-L1 expression in 187 cases found that PD-L1 TPS ≥ 1% was in 59.9% cases, while PD-L1 CPS ≥ 1% was in 72.2% cases. Furthermore, in 23 cases CPS was positive despite negative TPS score [45]. Moreover, when evaluating PD-L1 expression in 4459 advanced NSCLC cases, both TC and IC were considered, revealing predominant PD-L1 expression on immune cells. From all the positive PD-L1 expression cases, in 33% patients positive PD-L1 expression was restricted to IC, 6% to TC, and 26% of patients had PD-L1 expression on both TC and IC [46].

The contrast between prospective and retrospective studies results may be due to PD-L1 positivity decrease in archival compared to fresh tumour samples. However, in the FIR study of advanced or metastatic NSCLC, high agreement of high PD-L1 expression (TC ≥ 50% or IC  ≥ 10%) was observed between paired archival and fresh tumour samples [47]. Likewise, other clinical research of pembrolizumab in advanced NSCLC also demonstrated that PD-L1 expression levels evaluated by TPS were similar between archival and newly collected tumour specimens [48]. Similar results were seen in another study that contained not only advanced, but also stage I–III NSCLC, where no significant differences of PD-L1 expression rates between archival and fresh tumour specimens were detected [49]. However, according to recommendations, immunohistochemistry, including PD-L1 expression in older than 5 years specimens, should be discouraged [50]. In our study, this limit has not been exceeded.

These results lead to the hypothesis that higher PD-L1 expression is more likely to be found in advanced or metastatic NSCLC compared to early-stage NSCLC. This can be explained by the mechanism of PD-L1 pathway. For example, PD-L1 combined with PD-1 reduces the proliferation of PD-1 positive cells, inhibits their cytokine production, and induces apoptosis, inducing tumour immune escape [51]. The higher PD-L1 expression, the stronger attenuation of the host anti-tumour immune response, the better conditions for tumour growth. What is more, mice tumour models revealed that PD-L1 expression on either tumour cells or tumour-infiltrating immune cells contributes to tumour escape, but the contribution depends on TME [52]. A number of clinical trials demonstrated that positive and high PD-L1 expression is associated with larger tumour size, nerve and blood vessel invasion, as well as lymph node metastasis in NSCLC and other location malignancies [53,54]. On the other hand, other studies did not show statistically significant differences between the stage of NSCLC and PD-L1 expression, hence the results remain controversial [55].

Based on Blueprint phase 1 and Blueprint phase 2 project results, when assays with corresponding platforms and antibodies were compared (Ventana SP142, Ventana SP263, Dako 22C3, Dako 28-8, Dako 73-10), high agreement between assays was seen when PD-L1 expression was assessed on tumour cells, except SP-142 PD-L1 assay, which stained less tumour cells. Furthermore, positive PD-L1 expression on immune cells was observed in all assays, but with poor concordance among the assays [27,56]. However, in these studies, concordance between TPS and CPS was not assessed. What is more, later smaller studies evaluating PD-L1 expression in NSCLC reported high agreement between PD-L1 TPS and CPS [57]. In NSCLC, patients are selected for immunotherapy after assessment of PD-L1 expression as TPS, except the Ventana PD-L1 SP142 Assay [27,28]. Conversely, other cancers, such as head and neck squamous cell carcinoma, urothelial carcinoma, cervical cancer, and others PD-L1 expression is evaluated by IC or CPS [29,30].

In this study, it was found that PD-L1 TPS, IC, and CPS correlated with each other. There was almost perfect agreement when PD-L1 was evaluated as positive or negative. While there are no such data in resected NSCLC, clinical trials in other cancers or advanced NSCLC were conducted to evaluate whether different scoring systems, such as TPS, IC, and CPS yield concordant result. According to Guo et al., when PD-L1 expression was assessed using FDA approved 22C3 IHC assay by TPS, CPS, and IC, concordance between these three scoring methods was observed in breast cancer [58]. Furthermore, in study of advanced NSCLC, when the same IHC assay was used and PD-L1 was assessed by TPS and CPS, high agreement between these two scoring methods was detected (Kappa coefficient for adequacy was 0.82) [57]. However, the question if one scoring method could replace the other evaluating PD-L1 expression in NSCLC is still unanswered, further studies are required.

The results of this study determined that smoking intensity was significantly correlated with PD-L1 expression evaluated by TPS, IC, and CPS. Furthermore, smoking intensity gradually increased from no to high IC as well as CPS, and the significant difference was driven by PD-L1 high expressors. Both results showed that smoking intensity had stronger correlation with PD-L1 expression evaluated by IC compared to PD-L1 expression evaluated by TPS. This is in agreement with Song et al., who evaluated PD-L1 expression by TC and IC in histological material of 305 patients diagnosed with stage I–IV NSCLC. The results revealed that high PD-L1 expression is associated with smoking status and intensity [59]. Norum et al., in a systematic review, where nine papers on NSCLC were analysed, determined that high PD-L1 expression correlated with current or former smoking history in only three studies [60]. All these three studies were retrospective and only one study evaluated PD-L1 expression in resected specimens of stage I–III NSCLC. However, prospective studies, which included advanced or metastatic NSCLC and evaluated fresh biopsy samples, found no significant association between PD-L1 expression and smoking status [4,10,60,61,62,63,64,65]. In other malignancies, such as head and neck squamous cell carcinoma and human papillomavirus (HPV) positive oropharyngeal squamous cell carcinoma PD-L1 expression evaluated by TPS or CPS was significantly lower in current smokers [66,67]. The weight of evidence indicates that tobacco smoke has a diverse immunosuppressive or pro-inflammatory effect, resulting in TME changes [68]. Furthermore, some studies show that smokers demonstrate a better response when treated with anti-PD-1 or anti-PD-L1 monotherapy [69,70].

Zhu et al. revealed that in vitro, α5 nicotinic acetylcholine receptor (α5-nAChR), activated by exogenous compounds such as nicotine, induces PD-L1 expression in lung adenocarcinoma cell lines [71]. Moreover, in experimental in vitro and in vivo model, Wang et al. confirmed that one of the main tobacco carcinogens, benzo(a)pyrene (BaP), induces PD-L1 expression on lung epithelial cells, a process mediated by the Aryl hydrocarbon receptor (AhR) binding directly to the promoter of PD-L1 at −700 to −100 (region 1) [72]. Narayanapillai et al. showed that chronic inflammation that is characteristic for smokers diagnosed with COPD creates favourable conditions for immunosuppressive microenvironment with higher PD-L1 expression in tumour tissue, and precisely this type of tumours might have a greater benefit from immune checkpoint inhibitors. Furthermore, in mice, treated with the tobacco smoke carcinogen 4-(methylnitrosamino)-1- (3-pyridyl)-1-butanone (NNK) and lipopolysaccharide, the abundance of TAMs (M2 macrophages phenotype), PD-L1^+^ tumour cells, Foxp3^+^CD4^+^ T cells, and CD8^+^ T cells was higher compared with NNK-induced tumours [73].

Hence, studies regarding association between smoking and PD-L1 expression led to controversial results with minimal data on smoking intensity and PD-L1 expression on immune cells in TME in NSCLC. Our results suggest that higher smoking intensity induce higher PD-L1 expression, and evaluating PD-L1 expression by IC and CPS can be more specific looking for an association between PD-L1 expression and smoking.

In this study, it was also found that PD-L1 expression was associated with tumour histological type, revealing significantly higher PD-L1 TPS in squamous cell carcinoma compared with adenocarcinoma. Similar results were demonstrated in other study, where PD-L1 expression was significantly higher on tumour cells in squamous cell carcinoma compared to adenocarcinoma [74]. Although the exact mechanisms remain unknown, squamous cell carcinoma seems to be distinct from non-squamous cell carcinoma and recent genetic analyses showed that smoking related cancer, such as squamous-cell carcinoma, has larger TMB than NSCLC (mostly adenocarcinoma) in never-smokers [75]. Higher TMB hypothetically could induce higher T cells immune response and it could lead to higher PD-L1 expression on squamous cell carcinoma cells, respectively, to suppress these T cells. Some studies show that NSCLC with higher TMB is more sensitive to immunotherapy with PD-1/PD-L1 inhibitors [76]. Moreover, genetic/epigenetic alterations could affect PD-L1 expression as well. For example, in squamous cell NSCLC elevated levels of histone deacetylases (HDAC), such as HDAC3 and HDAC6, are detected, that are reported to upregulate PD-L1 expression by STAT3 signalling pathway in melanoma, osteosarcoma, and pancreatic cancer [77,78]. Furthermore, PD-L1 expression in dendritic cells in the TME and PD-L1 transcription in tumour cells are elevated through increased interferon -γ (IFN-γ) production and acetylation of PD-L1 promoter region histone after HDAC3 expression inhibition [78].

Evaluating PD-L1 expression and TILs in the TME, significant correlation between PD-L1 IC and CPS, but not TPS and the number of tumour-infiltrating CD4^+^ T cells, Foxp3^+^CD4^+^ T cells, and CD8^+^ T cells were detected. PD-L1 IC > 1% was associated with higher CD4^+^ T cell infiltration level and PD-L1 CPS > 1%, with higher CD4^+^ T cells and CD8^+^ T cells infiltration level. Only PD-L1 IC and CPS > 10% was associated with higher Foxp3^+^CD4^+^ T cells infiltration level in tumour tissue.

Limited data exist regarding PD-L1 expression and TILs in early-stage NSCLC. Giatromanolaki et al. showed that PD-L1 TPS was significantly associated with TILs density, especially with tumour-infiltrating CD4^+^Foxp3^+^ T cells in 98 stage II–III resected NSCLC patients [79]. Results regarding the association between PD-L1 expression on tumour or immune cells and TILs in other malignancies are controversial. This discrepancy may be attributed to variations in tumour types, diverse TME, and different underlying mechanisms [80,81]. Similarly to our results, Albrecht et al. demonstrated that PD-L1 expression evaluated by IC positively correlated with CD8^+^ T cells as well as with CD4^+^ T cells in 131 gallbladder cancer patients [82]. Svensson et al. reported association between PD-L1 IC expression and CD4^+^Foxp3^+^ T cells in 148 resected tumour specimens from early to advanced stage oesophageal and gastric carcinoma [83]. In squamous bladder cancer, association was found between PD-L1 expression evaluated by CPS and CD8^+^ T cells, but not CD4^+^ T cells [80]. Although some authors suggest that IL-27 could induce PD-L1 expression on CD8^+^T cells, the mechanisms of interaction between PD-L1 expression and TILs remain unknown [84]. Similar to our results, Kleinovink et al., using murine colon carcinoma model, found that there was no significant difference of the myeloid immune infiltrate in tumours with PD-L1 positive or negative expression on tumour cells, demonstrating that TME is not strongly affected by PD-L1 expression on tumour cells. On the other hand, some data demonstrated that PD-L1 expression on immune cells can also contribute to immune evasion and tumour outgrowth. Ultimately, tumours with PD-L1 negative tumour cells still had positive effect of anti PD-1 therapy, demonstrating that the treatment effect could effectively mediated through the inhibition of PD-1/PD-L1 pathway on immune cells [81]. In most of haematological malignancies, such as lymphoma and in solid tumours, such as NSCLC, cancer cells express MHC II and become direct targets for CD4^+^ T cells [85,86,87]. Murine models demonstrated that anti PD-1 therapy exerted antitumour effect on MHC-I^−^MHC-II^+^ tumours in a cytotoxic CD4^+^ T cell-dependent manner, while the same effect on MHC-I^−^MHC-II^−^ tumours was not reached [88]. Francisco et al. in murine model revealed that PD-L1 expression on tumour cells plays a major role in regulating induced CD4^+^Foxp3^+^ T cells development, but this does not explain in what manner CD4^+^Foxp3^+^ T cells are associated with PD-L1 expression on immune cells [89]. Further studies have revealed that TILs, such as CD8^+^ T cells and CD4+ T cells, including Foxp3^+^CD4^+^ T cells subset, are known to induce PD-L1 expression on tumour and immune cells in a number of types of cancers, including NSCLC [79,90].

The results of this study revealed an association between PD-L1 expression and the infiltration of M2 macrophages in the tumour. When TPS was high, there was a bigger probability to detect a higher number of tumour-infiltrating M2 macrophages. In Sumitomo et al.’s work, where 160 cases of early-stage NSCLC patients were included and surgically resected tumour specimens were assessed, PD-L1 TC and IC were significantly higher in the tumour-infiltrating M2 macrophages group [26]. Although the exact mechanism in lung cancer is unknown, studies from other malignancies have demonstrated that tumour-infiltrating M2 macrophages can increase PD-L1 expression in tumour cells as well as in immune cells by secreting different cytokines. For example, upregulation may occur via tumour necrosis factor-α (TNF-α) through the nuclear factor kappa B (NF-κB) signalling pathway in pancreatic ductal carcinoma cells [91]. Additionally, in breast cancer, there’s evidence of alteration in metabolism to a glycolytic type through the transforming growth factor-β (TGF-β)-STAT1-succinate dehydrogenase (SDH)-hypoxia-inducible factor 1α (HIF1α) pathway, both in ex vivo and in vivo murine models [92]. It is known that high abundance of M2 TAMs in tumour tissue is often closely related to the occurrence of resistance to anti-PD-1/anti-PD-L1 therapy [93,94,95]. Our results further support the theory that M2 create a tumour promoting TME by inhibiting T cells activity and increasing expression of the PD-L1 in the TME.

This data confirms that TILs and M2 macrophages crosstalk in the TME affects the expression of PD-L1 on tumour cells and immune cells, leading to hypothesis that PD-L1 expression assessment in concern of TILs (CD4^+^ T cells, Foxp3^+^CD4^+^ T cells, and CD8^+^ T cells) and TAMs (M2 macrophages) may provide additional benefit selecting patients for immunotherapy.

We found that in our study PD-L1 is not a reliable indicator of prognosis in resected NSCLC. Most of the studies, where PD-L1 score was determined as prognostic or predictive factor, included advanced or metastatic cancer [18,19]. On the other hand, in early-stage (I–III) NSCLC patients who underwent surgical resection, neither PD-L1 expression nor TILs infiltration alone, nor their combination, was associated with patient’s prognosis [24]. This can lead to the assumption that in early-stage (I–III) NSCLC PD-L1 and TILs are not sufficiently reliable prognostic biomarkers. These controversial results can be due to pitfalls of PD-L1 staining and scoring. Various studies conduct immunohistochemistry using different assays, each with distinct thresholds for positivity and different specimen size (biopsy or resection specimens). Additionally, differences exist in the location of analysis (primary tumour and metastases). Due to tumour heterogeneity, inaccuracies in PD-L1 testing are inevitable when analysing small biopsies instead of resected specimens [96]. Moreover, the same tumour can demonstrate different PD-L1 expression and tumour-infiltrating immune cells density in primary tumour compared to metastatic tissue [97].

The limitations of our study include a small sample size and the utilisation of archival histological specimens. Further studies of PD-L1 expression on both tumour and immune cells within the context of the TME in early-stage NSCLC and unification of PD-L1 scoring methods are needed.

## 5. Conclusions

Our study demonstrated that rates of PD-L1 expression correlated among three scoring methods (TPS, IC, and CPS). We found a significant association between PD-L1 expression and smoking intensity, histological type of squamous cell carcinoma, and the infiltration of the immune cells: CD4^+^ T cells, Foxp3^+^CD4^+^ T cells, CD8^+^ T cells, and M2 macrophages, in the tumour microenvironment.

## Figures and Tables

**Table 1:** Characteristics of study population.

Baseline Characteristics	n (Total) = 72
Gender, n (%)	
Male	58 (80.6)
Female	14 (19.4)
Age group, n (%)	
<65 years	33 (45.8)
≥65 years	39 (54.2)
Smoking status, n (%)	
Non-smokers	13 (18.1)
Current and former smokers	59 (81.9)
Pack-years, median (range)	30 (0–60)
COPD, n (%)	
Absent	50 (69.4)
Present	22 (30.6)
Histological NSCLC type, n (%)	
Adenocarcinoma	36 (50)
Squamous cell carcinoma	30 (41.7)
Large cell carcinoma	6 (8.3)
Differentiation, n (%)	
Well-moderate	39 (54.2)
Poor-undifferentiated	33 (45.8)
NSCLC stage, n (%)	
IA-IB	20 (27.8)
IIA-IIB	24 (33.3)
IIIA-IIIB	28 (38.9)
pT status, n (%)	
pT1	12 (16.7)
pT2	41 (56.9)
pT3-4	19 (26.4)
Lymph node status, n (%)	
pN0	31 (43.1)
pN1	23 (31.9)
pN2	16 (22.2)
pN3	2 (2.8)
Adjuvant therapy, n (%)	
Given	31 (43.1)
Not given	41 (56.9)

**Table 2:** Characteristics of NSCLC patients with positive and negative PD-L1 expression subgroup.

	PD-L1 Expression					
	TC		IC		CPS	
	Positive	Negative	Positive	Negative	Positive	Negative
**Gender, n (%)**						
**Male**	17 (29.3)	41 (70.7)	22 (37.9)	36 (62.1)	24 (41.4)	34 (58.6)
**Female**	3 (21.4)	11 (78.6)	4 (28.6)	10 (71.4)	4 (28.6)	10 (71.4)
**Age group, n (%)**						
**<65 years**	8 (24.2)	25 (75.8)	10 (30.3)	23 (69.7)	10 (30.3)	23 (69.7)
**≥65 years**	12 (30.8)	27 (69.2)	16 (41)	23 (59)	18 (46.2)	21 (53.8)
**Smoking status, n (%)**						
**Non smokers**	2 (15.4)	11 (84.6)	2 (15.4)	11 (84.6)	2 (15.4)	11 (84.6)
**Former smokers and smokers**	18 (30.5)	41 (69.5)	24 (40.7)	35 (59.3)	26 (44.1)	33 (55.9)
**COPD, n (%)**						
**Absent**	12 (24)	38 (76)	17 (34)	33 (66)	19 (38)	31 (62)
**Present**	8 (36.4)	14 (63.6)	9 (40.9)	13 (59.1)	9 (40.9)	13 (59.1)
**Histological NSCLC type, n (%)**						
**Adenocarcinoma**	7 (19.4)	29 (80.6) *	10 (27.8)	26 (72.2)	11 (30.6)	25 (69.4)
**Squamous cell carcinoma**	13 (43.3)	17 (56.7)	14 (46.7)	16 (53.3)	15 (50)	15 (50)
**Differentiation, n (%)**						
**Well-moderate**	13 (33.3)	26 (66.7)	12 (30.8)	27 (69.2)	14 (35.9)	25 (64.1)
**Poor**	7 (21.2)	26 (78.8)	14 (42.4)	19 (57.6)	14 (42.4)	19 (57.6)
**NSCLC stage, n (%)**						
**IA-IB**	7 (30.4)	16 (69.6)	5 (21.7)	18 (78.3)	7 (30.4)	16 (69.6)
**IIA-IIB**	8 (34.8)	15 (65.2)	12 (52.2)	11 (47.8)	12 (52.2)	11 (47.8)
**IIIA-IIIB**	5 (19.2)	21 (80.8)	9 (34.6)	17 (65.4)	9 (34.6)	17 (65.4)
**pT status, n (%)**						
**pT1**	3 (25)	9(75)	5 (41.7)	7(58.3)	5 (41.7)	7 (58.3)
**pT2**	10 (24.4)	31 (75.6)	12 (29.3)	29 (70.7)	14 (34.1)	27 (65.9)
**pT3-4**	7 (36.8)	12 (63.2)	9 (47.4)	10 (52.6)	9 (47.4)	10 (52.6)
**Lymph node status, n (%)**						
**Negative (pN0)**	8 (25.8)	23 (74.2)	9 (29)	22 (71)	10 (32.3)	21 (67.7)
**Positive (pN1-N3)**	12 (29.3)	29 (70.7)	17 (41.5)	24 (58.5)	18 (43.9)	23 (56.1)

* *p* < 0.05, chi-square (χ^2^) test.

**Table 3:** Association between positive and negative PD-L1 expression subgroup and immune cells infiltration level in tumour tissue.

	PD-L1 Expression					
	TPS		IC		CPS	
	Positive	Negative	Positive	Negative	Positive	Negative
**CD4^+^T cells n (%)**						
**low**	8 (21.1)	30 (78.9)	9 (23.7)	29 (76.3) *	10 (26.3)	28 (73.7) *
**high**	12 (35.3)	22 (64.7)	17 (50)	17 (50)	18 (52.9)	16 (47.1)
**CD8^+^T cells n (%)**						
**low**	7 (22.6)	24 (77.4)	8 (25.8)	23 (74.2)	8 (25.8)	23 (74.2) *
**high**	13 (31.7)	28 (68.3)	18 (43.9)	23 (56.1)	20 (48.8)	21 (51.2)
**Foxp3^+^CD4^+^T cells n (%)**						
**low**	8 (25)	24 (75)	8 (25)	24 (75)	9 (28.1)	23 (71.9)
**high**	12 (30)	28 (70)	18 (45)	22 (55)	19 (47.5)	21 (52.5)
**IL17A^+^CD4^+^T cells n (%)**						
**low**	11 (27.5)	29 (72.5)	16 (40)	24 (60)	17 (42.5)	23 (57.5)
**high**	9 (28.1)	23 (71.9)	10 (31.3)	22 (68.8)	11 (34.4)	21 (65.6)
**M1 macrophages, n (%)**						
**low**	10 (27.8)	26 (72.2)	13 (36.1)	23 (63.9)	15 (41.7)	21 (58.3)
**high**	10 (27.8)	26 (72.2)	13 (36.1)	23 (63.9)	13 (36.1)	23 (63.9)
**M2 macrophages, n (%)**						
**low**	4 (17.4)	19 (82.6)	8 (21.7)	18 (78.3)	5 (21.7)	18 (78.3)
**high**	6 (27.3)	16 (72.7)	5 (22)	17 (77.3)	7 (31.8)	15 (68.2)

* *p* < 0.05, chi-square (χ^2^) test.

**Table 4:** Association between positive and negative PD-L1 expression subgroup and the number of tumour-infiltrating immune cells.

	PD-L1 Expression					
	TPS		IC		CPS	
	Positive	Negative	Positive	Negative	Positive	Negative
CD4^+^ T cells	224.5 (116-538)	279 (134–504)	210 (116–522)	285.5 (126–538) *	210.5 (116–522)	279 (126–538) *
CD8^+^ T cells	256 (92–405)	265 (109–406)	250.5 (92–405)	268.5 (109–406)	248.5 (92–405)	272.5 (109–406)
Foxp3^+^CD4^+^ T cells	56.5 (13–92)	57.5 (16–90)	53 (13–92)	58 (16–90)	53 (13–92)	58 (16–90)
IL-17A^+^CD4^+^ T cells	23 (11–46)	22.5 (13–47)	23.5 (11–46)	22 (12–47)	23.5 (11–46)	22 (12–47)
M1 macrophages	100.5 (54–171)	102 (64–147)	100.5 (54–171)	102 (58–147)	102 (54–171)	97.5 (58–147)
M2 macrophages	101 (45–153)	102 (64–147)	104 (45–153)	106.5 (82–186)	101 (45–153)	110 (82–186)

The number of tumour-infiltrating immune cells represents median (range) per ten high-power fields in tumour tissue. * *p* < 0.05 Mann-Whitney U test.

**Table 5:** Association between high and non-high PD-L1 expression subgroup and the number of tumour-infiltrating immune cells.

	PD-L1 Expression					
	TPS		IC		CPS	
	<50%	≥50%	<10%	≥10%	<10%	≥10%
CD4^+^ T cells	230 (116–538)	396 (134–425)	231.5 (116–522)	283 (177–538)	215.5 (116–522)	285.5 (134–538)
CD8^+^ T cells	259 (92–405)	272 (230–406)	266 (92–406)	245.5 (123–391)	261.5 (92–405)	259 (109–406)
Foxp3^+^CD4^+^ T cells	57 (13–92)	67 (26–77)	53 (13–92)	65.5 (55–90) *	53 (13–92)	62 (26–90) *
IL-17A^+^CD4^+^ T cells	23 (11–47)	27 (14–42)	23 (11–46)	21 (13–47)	23 (11–46)	21.5 (13–47)
M1 macrophages	100 (54–171)	118 (74–147)	105.5 (54–171)	81.5 (64–128)	102 (54–171)	97.5 (64–147)
M2 macrophages	104 (45–153)	168 (150–186) *	106 (45–186)	-	106 (45–153)	112 (85–186)

The number of tumour-infiltrating immune cells represents median (range) per ten high-power fields in tumour tissue. * *p* < 0.05 Mann-Whitney U test.

## Data Availability

The data presented in this study are available on request from the corresponding author.
